# A tale of two sisters – delayed diagnosis of genetic hyperinsulinaemic hypoglycaemia

**DOI:** 10.1530/EDM-24-0007

**Published:** 2024-08-16

**Authors:** F Stringer, C Preston, R MacIsaac, F Inchley, L Rivera-Woll, S Farrell, N Sachithanandan

**Affiliations:** 1 Departments of Endocrinology and Diabetes, Surgery and General Medicine, St Vincent’s Hospital Melbourne and the University of Melbourne, Victoria, Australia; 2 Western Health, Melbourne, Victoria, Australia; 3 Australian Centre for Accelerating Diabetes Innovations, University of Melbourne, Victoria, Australia; 4 Endocrinology Melbourne, Victoria, Australia

**Keywords:** Adult, Female, White, Australia, Pancreas, Diabetes, Hyperinsulinaemic hypoglycaemia, Unique/unexpected symptoms or presentations of a disease, August, 2024

## Abstract

**Summary:**

Congenital hyperinsulinism is the leading cause of persistent hypoglycaemia in infants and children; however, it is uncommon to be diagnosed in adulthood. We describe the cases of two sisters who presented with hyperinsulinaemic hypoglycaemia aged 47 and 57 years old, who were subsequently diagnosed with compound heterozygous likely pathogenic variants in the *ABCC8* gene, a known cause of monogenic congenital hyperinsulinism. We discuss the typical presenting features, investigation findings, and treatment strategies for patients with this condition.

**Learning Points:**

- Congenital hyperinsulinism is a rare cause of hyperinsulinaemic hypoglycaemia diagnosed in adulthood.
- Clinical presentation is similar to an insulinoma, and imaging modalities may assist in differentiation.
- There are minimal medical therapies currently available for patients non-responsive to diazoxide (such as those with *ABCC8* and *KCNJ11* variants).
- Continuous glucose monitoring can be helpful in giving patients autonomy in managing their disease, as well as relieving anxiety and fear associated with hypoglycaemia.

## Background

Congenital hyperinsulinism is a rare heritable condition that causes persistent hypoglycaemia. Presentation and diagnosis typically occur during infancy and childhood and portend a significant risk of neurological complications. Rarely does the condition present in adulthood.

Here, we report a case of hyperinsulinaemic hypoglycaemia diagnosed in an otherwise well woman at the age of 47 years. Interestingly, her older sister had a significant history for intellectual impairment following severe hypoglycaemia at birth. Both sisters were subsequently discovered to have novel compound heterozygous variants in the *ABCC8* gene, consistent with autosomal recessive monogenic hyperinsulinism.

## Case presentation

A 47-year-old woman presented with increasing frequency and severity of hypoglycaemic episodes. These were characterised by typical symptoms of light-headedness, difficulty concentrating, irritability and headache, and occurred both fasting and post-prandially. She noted these symptoms improved with oral intake and self-treated by consuming carbohydrates every 1–2 h, resulting in significant weight gain of over 15 kg.

Her past medical history included pernicious anaemia and asthma, as well as documented transient neonatal hypoglycaemia followed by normal developmental milestones throughout childhood and adolescence. Her family history was significant for an older sister with intellectual impairment presumed to be secondary to severe hypoglycaemia at birth, with recent worsening of hypoglycaemic seizures. Her mother had died from an unknown malignancy, and her father and younger sister were medically well. One maternal aunt had died within hours of birth.

## Investigation

Hyperinsulinaemic hypoglycaemia was confirmed on a 72-h fast, with normal baseline cortisol and IGF-1, and the fast aborted early at 60 h at a laboratory plasma glucose of 2.0 mmol/L, with a corresponding inappropriately elevated insulin level of 4 mU/L and C-peptide of 0.49 pmol/mL, with a beta hydroxybutyrate level of 2.97 mmol/L. She had a significant response to 1 mg IV glucagon at the conclusion of the test, with her plasma glucose increasing from 2.0 mmol/L to 4.5 mmol/L.

Similarly, the patient’s sister also had hyperinsulinaemic hypoglycaemia confirmed on a 72-h fast, aborted after 66 h at a laboratory plasma glucose of 1.8 mmol/L, with inappropriately elevated insulin of 4 mU/L and C-peptide of 0.44 pmol/mL, and a beta-hydroxybutyrate level of 0.73 mmol/L. CT pancreas and gallium-68 DOTATATE PET were unable to localise an insulinoma.

**Figure 1:**
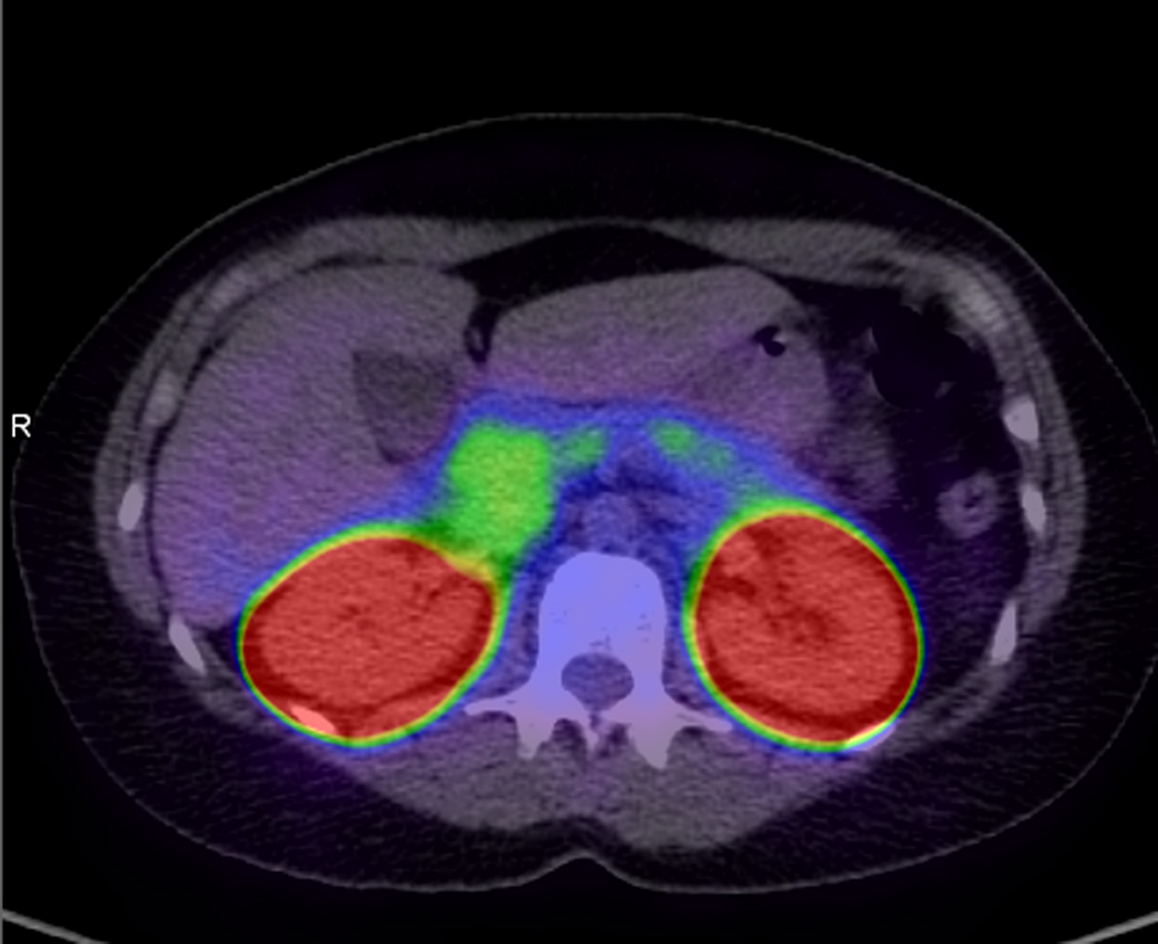
Ga-68 GLP-1 PET-CT demonstrating diffuse GLP-1 uptake in an enlarged pancreatic tail.

An endoscopic ultrasound identified an inconclusive lesion in the pancreatic tail; however, a subsequent gallium-68 DOTA-exendin-4 PET-CT (Ga-68 GLP-1 PET) demonstrated marked uptake in the entire pancreatic tail, consistent with diffuse nesidioblastosis rather than a discrete lesion compatible with insulinoma (Fig. 1). Interestingly, this test was complicated by profound hypoglycaemia (BGL: 1.7 mmol/L) requiring intravenous dextrose.

**Figure 2:**
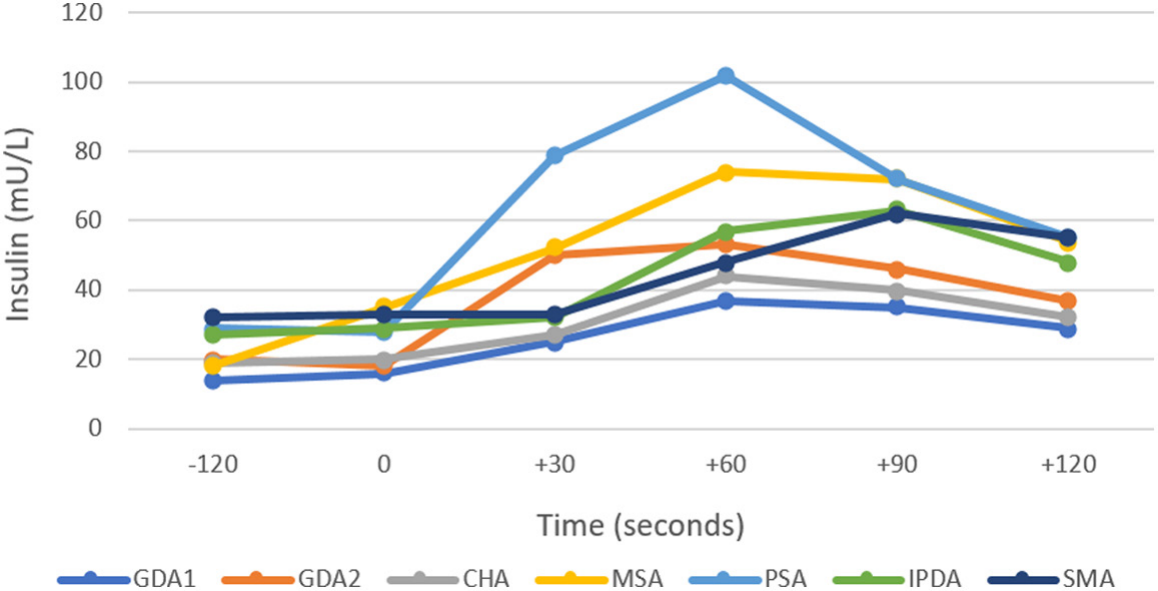
Calcium stimulation test demonstrating mild global response in insulin secretion following intra-arterial calcium injection. CHA, common hepatic artery; GDA1, mid gastroduodenal artery; GDA2, proximal gastroduodenal artery with accessory right hepatic artery; IPDA, inferior pancreaticoduodenal artery; MSA, mid splenic artery; PSA, proximal splenic artery; SMA, superior mesenteric artery.

Due to the incongruent results, the patient proceeded to a calcium stimulation test, which demonstrated a mild two- to four-fold global rise in insulin in response to calcium across all arterial territories, consistent with the diffuse increased uptake on Ga-68 GLP-1 PET (Fig. 2).

Given these results, coupled with her family history, genetic testing was undertaken and revealed compound heterozygous variants in the *ABCC8* gene, with one variant (missense) already classified as likely pathogenic (c.2522G>A, p.Arg841gln) and the second as a new missense variant of unknown significance (c.4595T>C, p.Val1532Ala). Cascade testing of the affected sister revealed the same variants. Testing of their unaffected father confirmed paternal inheritance of the known variant, and an unaffected sister had the second variant, indicating maternal inheritance. The new variant has now been reclassified as likely pathogenic, with the sisters’ genotype therefore consistent with autosomal recessive ABCC8-associated hyperinsulinism.

## Treatment

Dietary modification formed the mainstay of non-pharmacological treatment, with frequent long-acting carbohydrate-containing meals and increased fibre intake. Formal dietitian reviews were arranged to assist with ongoing management.

Diazoxide was initially trialled at 50 mg three times daily; however, it was self-ceased within a week due to the increased frequency of hypoglycaemia. Acarbose was then trialled at 50 mg three times daily; however, it was also ceased due to intolerable gastrointestinal side effects. Low-dose verapamil was considered but not trialled due to the patient’s fear of potential side effects.

Eventually, self-funded continuous glucose monitoring (CGM) proved beneficial in alleviating the anxiety associated with hypoglycaemia and, combined with ongoing dietary modification, has been extremely successful in the avoidance of major hypoglycaemic events.

## Outcome and follow-up

The patient is now 50 years of age and has been followed from 2018 to the present. At the last review, she was continuing to do well with minimal hypoglycaemic events, aided by the assistance of CGM informing her ongoing dietary modifications.

Her sister lives in a residential aged care facility due to her intellectual impairment and is also doing well with CGM and dietary modification. She has had no further major hypoglycaemic events.

## Discussion

Congenital hyperinsulinism (CHI) is the leading cause of persistent hypoglycaemia in infants and children, characterised by dysregulated insulin secretion from pancreatic β-cells despite low glucose levels (1). The causes can be broken down into three distinct subgroups: transient due to perinatal stress, monogenic forms due to single gene defects, and syndromic forms, e.g. Beckwith–Wiedemann syndrome.

Our case involved a monogenic cause of hyperinsulinism, which affects 1/50 000 live births, with the most severe forms being caused by inactivating mutations of the *ABCC8* and *KCNJ11* genes (1, 2) (that account for 36–70% of congenital hyperinsulinism cases) (3). *ABCC8* and *KCNJ11* genes encode the two subunits (the sulphonylurea receptor 1 (SUR1) and the inwardly rectifying potassium channel 6.2 (KIR6.2), respectively) of the β-cell potassium–ATP channel (ATP-sensitive K+ channel (K-ATP)) (2), which is critical in controlling glucose-mediated insulin secretion from pancreatic β-cells.

In the normal β-cell, closure of this channel, following the increase of the ATP/ADP ratio resulting from the phosphorylation of glucose, leads to membrane depolarisation and a rise in free intracellular Ca^2+^ concentration from activation of the voltage-gated calcium channels, which in turn triggers insulin secretion. Defects in the trafficking of the channel to the cell surface or channel dysfunction lead to dysregulated insulin secretion independent of glucose levels (2, 3). Patients with congenital hyperinsulinism (CHI) typically present in early infancy with seizures, coma, and failure to thrive, with severe fasting hypoglycaemia with up to half of affected children having adverse neurodevelopment (1).

There are two distinct histological subtypes of CHI due to potassium–ATP channel dysfunction. In focal disease (affecting 30–40% of patients), there is a discrete area of β-cell proliferation. This is explained by the ‘two-hit hypothesis’ whereby patients inherit a recessive variant in the *ABCC8* or *KCNJ11* gene from their unaffected father and then have a somatic loss of heterozygosity (LOH) of the second unaffected maternal allele (maternal 11p 15.5 somatic loss) in the affected β-cells. This LOH results in an imbalance between genes controlling cell proliferation, high expression of IGF2, and β-cell hyperplasia, while the complete loss of either *ABCC8* or *KCNJ11* leads to inappropriate insulin secretion. In diffuse disease (affecting 60–70% of patients), there is hyperactivity of β-cells throughout the entire endocrine pancreas. This is caused by biallelic recessively inherited loss of function variants, or less commonly, dominantly inherited variants (4).

Investigations in patients with persistent CHI will reveal hyperinsulinaemic hypoglycaemia, with blood glucose levels <3 mmol/L and inappropriate C-peptide and insulin levels. Gallium-68 DOTA-Exendin-4 PET-CT (Ga-68 GLP-1 PET) has been shown to be highly specific and sensitive for diagnosing insulinomas by binding to the GLP-1R on β-cells in the pancreas. In a small prospective study of patients with focal CHI pre-surgery, Ga-68 GLP-1 PET had higher sensitivity than the previously used 18F-DOPA PET (5), indicating superiority in diagnosing this condition.

CHI does not have a consistent trajectory. Homozygous and compound heterozygous mutations are more likely to suggest permanent disease, but disease severity can decrease over time (3). A recently published ambispective study followed 18 patients with *ABCC8* variants for a mean duration of 17 years. This study showed that 12 patients had spontaneous resolution of hypoglycaemia, with five patients having progression to diabetes with insufficient insulin secretion. The above study also reported a higher percentage of patients progressing to diabetes who presented with biallelic variants than those who did not, although this difference was not statistically significant. Hypotheses proposed to explain these findings include a reduction in β-cells due to progressive apoptosis from high levels of intracellular calcium, or due to a change in gene expression and alteration in metabolic pathways (6).

The aim of treatment in CHI is to stabilise blood glucose levels, prevent neuroglycopenia, and long-term neurological sequelae. Diazoxide, a commonly used treatment in patients with hypoglycaemia, works by opening K-ATP channels on the β-cells, causing re-polarisation of the membrane, and thereby leading to a decrease in insulin secretion. However, due to this mechanism of action, homozygous and compound heterozygous recessive mutations in the *ABCC8* gene (as seen in this case), which affect the potassium channel resulting in permanent channel closure, are unresponsive to diazoxide (2).

Second-line therapy includes the use of octreotide or other somatostatin analogues. These bind to somatostatin receptors on β-cells and decrease the formation of cAMP, thus reducing insulin secretion (8). Patients can develop tachyphylaxis to these medications due to β-cell internalisation of receptors, but studies have shown stabilisation of blood glucose levels and improvement in patients’ quality of life (7, 8). Other potential treatment options include calcium channel receptor antagonists such as nifedipine or verapamil. These reduce calcium-mediated exocytosis of insulin from β-cells; however, a small study that trialled escalating doses of nifedipine in children with mutations in the *ABCC8* gene showed nil benefit (7). Acarbose can be used when dietary modifications fail to prevent post-prandial hypoglycaemia, by slowing glucose absorption to decrease the glycaemic peak followed by insulin release (3).

More recently, mTOR inhibitors such as sirolimus and everolimus have been used in patients with CHI unresponsive to other medical therapies. The intracellular mTOR pathway is involved in β-cell growth and altered insulin secretion in patients with insulinoma, ketone body synthesis, and insulin action. However, efficacy is variable, and due to its immunosuppressive side effects, it is not routinely recommended for long-term use (9). The glucose dependency of GLP-1 is lost in patients with CHI from K-ATP variants, which would explain the severe hypoglycaemia experienced by our patient during her Ga-68 GLP-1 PET (10). Of promise, GLP-1 receptor antagonists have been shown to ameliorate insulin secretion in *in vitro* and *in vivo* models of CHI due to K-ATP channel mutations and may become a viable therapeutic option in the future, pending confirmation in human studies (10).

Given these somewhat limited pharmacological options, the combination of dietary changes, education, and CGM is the mainstay of treatment in many patients. Dietary modifications include frequent feeding, long-acting carbohydrates, abundant protein, fibre supplements, and fat emulsions. These aim to prevent a sudden rise in blood glucose that would cause a rapid insulin release and subsequent hypoglycaemia (3, 7). CGM allows patients autonomy to manage their disease, as well as relieving anxiety associated with unpredictable hypoglycaemia. This has proved invaluable for our patients, with a significantly decreased burden of disease and improvement in quality of life.

Hypoglycaemic episodes that cannot be eliminated with the use of maximum tolerated doses of medication are an indication to consider surgical treatment, especially in the focal form of the disease. However, in the diffuse form, lesions are located throughout the pancreas, and patients may require near-total pancreatectomy (95–98% removal), with complications of diabetes, ongoing CHI, pancreatic exocrine insufficiency and damage to the common bile duct (2, 4) and surgery is best avoided.

This case demonstrates the challenges associated with diagnosing and managing patients with congenital hyperinsulinism. It highlights that the diagnosis of CHI can be delayed into adulthood and that clinicians need to maintain a high index of suspicion when the history or investigations for hypoglycaemia are suggestive. The lack of efficacious and safe agents in managing CHI due to K-ATP channel mutations was overcome by patient education, dietary modification and the use of CGM.

## Declaration of interest

The authors declare that there is no conflict of interest to declare that could be perceived as prejudicing the impartiality of the study reported.

## Funding

This work did not receive any specific grant from any funding agency in the public, commercial or not-for-profit sector.

## Patient consent

Written informed consent for publication of their clinical details was obtained from the patient.

## Author contribution statement

FS wrote up the case report and body of the discussion. CP and NS were involved in making revisions and improvements to the article. FI, LR, RM and SF are involved in the clinical care of the patient. All authors approved the final submitted version of the manuscript.
